# Status of Biofilm Production and Vancomycin Resistance in Enterococcus in the Rural Population of Mathura, India

**DOI:** 10.7759/cureus.43351

**Published:** 2023-08-11

**Authors:** Dinesh Kumar, Priya Mehrishi, Sameer Singh Faujdar, Bajarangi Lal Chaudhary, Sonu Panwar

**Affiliations:** 1 Microbiology, Krishna Mohan Medical College & Hospital, Mathura, IND; 2 Microbiology, Maharishi Markandeshwar Medical College and Hospital, Solan, IND; 3 Microbiology, Hind Institute of Medical Sciences, Mau, IND

**Keywords:** enterococcus faecium, enterococcus faecalis, vancomycin-resistant enterococci, enterococcus, biofilm producer

## Abstract

Introduction

Hospital-acquired or nosocomial infections caused by the rapidly emerging bacteria vancomycin-resistant enterococci can be dangerous and even fatal. Therefore, this study aimed to investigate the presence of enterococci in various clinical specimens along with their vancomycin resistance status and biofilm-producing capabilities.

Methods

A total of 164 *Enterococcus* species were isolated and further included in this study. Isolation and identification were done by the standard bacteriological procedure, antibiotic susceptibility testingwas done by clinical laboratory standard guidelines, and biofilm production test was done by microtiter plate methods.

Results

Among the total of 164 isolates, *Enterococcus faecalis* constituted 60.97% and *Enterococcus faecium* constituted 39.02%. Maximum isolates were from urine samples. The prevalence of vancomycin-resistant *Enterococcus* was 6.70%, and 18.29% of *Enterococcus* isolates were biofilm producers. The sensitivity among the biofilm producers was maximum for linezolid (87.33%), followed by teicoplanin (86.43%) and vancomycin (79.64%).

Conclusion

High prevalence of enterococci was found in urine samples and* *biofilm producers *Enterococcus *isolates were more antibiotic-resistant than non-biofilm producers.

## Introduction

Enterococci are microbiota of humans and animals. Enterococci live as normal flora of the gastrointestinal (GI) tract, biliary tract, urethra, and vagina. *Enterococcus* cause a wide range of infections in humans. These infections are skin and soft tissue infection, urinary tract infection (UTI), intra-abdominal infection, pelvic infection, bacteremia, meningitis, infective endocarditis, and neonatal sepsis. Enterococci are the third most common agents of nosocomial UTIs. Due to their antibiotic resistance, enterococci are also becoming more and more significant disease-causing organisms in humans, particularly in hospitals [1,2]. Because of the organism’s great ability to develop resistance to antibiotics such as penicillin, vancomycin, and concentrations of aminoglycosides, serious enterococcal infections are often challenging to treat [3]. There is intrinsic resistance within the *Enterococcus* species to commonly used antibiotics including aminoglycosides and beta-lactams, and in actuality, the therapeutic efficacy of clindamycin, trimethoprim/sulfamethoxazole, cephalosporins, and aminoglycosides in controlling *Enterococcus *is questionable. Significant concern has been raised about enterococci that are vancomycin-resistant and their effects in nosocomial infections. Among the concerns raised, the major one is the emergence of acquired vancomycin resistance in clinical enterococcal isolates [4,5]. Additionally, the ability of Enterococci especially *E. faecalis *and *E. faecium* to produce biofilm increases their resistance to antibiotics and increases their capacity to cause disease [6]. In hospitals, biofilms are communities of microorganisms attached to biological or non-biological surfaces and encased in extracellular matrix [7,8]. The cells of the microorganisms that form a biofilm are in close contact, which increases the sharing of their unique traits. Microbial cells developing in a biofilm are therefore biologically distinct from free-flattened bacteria. Antimicrobial resistance and the development of biofilm make them challenging to get treatment [4,9]. Therefore, in this research, *Enterococcus* species isolated from distinct clinical specimens were assessed for their capacity to form biofilms and their patterns of antibiotic-susceptibility including vancomycin-resistant enterococci (VRE).

## Materials and methods

The current research was conducted in the Microbiology Department of K. D. Medical College, Mathura, Uttar Pradesh, India. All clinical samples received from January to June 2018 were included in the study, and the study was approved by Institutional Ethics Committee. The inclusion criterion for the current study was that all gram-positive catalase-negative cocci isolated from various clinical samples were considered, and gram-positive cocci (catalase-positive) and all gram-negative bacteria were excluded. All clinical specimens (urine, blood, pus, sputum, vaginal swabs, and aspirates) received in the laboratory were cultured for the isolation of *Enterococcus* species. *Enterococcus* species were identified by standard biochemical procedures such as aesculin hydrolysis, acid production from sugars, and PYRase and NH3 production from arginine [10]. Susceptibility tests to antibiotics were performed by the Kirby-Bauer technique, and zones of inhibitions were interpreted in accordance to the CLSI (Clinical and Laboratory Standards Institute) guidelines [11,12]. Penicillin (10 units), ampicillin (10 µg), erythromycin (15 µg), tetracycline (30 µg), ciprofloxacin (5 µg), chloramphenicol (30 µg), teicoplanin (30 µg), nitrofurantoin (300 µg), levofloxacin (5 µg), norfloxacin (10 µg), rifampin (5 µg), linezolid (30 µg), and vancomycin (30 µg) were checked for their susceptibility.

Detection of high-level aminoglycoside resistance

The disc-diffusion method was used to detect high-level aminoglycoside resistance using discs containing high-level gentamicin (120 µg) and high-level streptomycin (300 µg). Plates were incubated, and results were read after 24 hours. When the zone diameter was greater than 10 mm, *Enterococcus *was considered susceptible to aminoglycosides, whereas a zone diameter of 6 mm indicated resistance and 7-9 mm indicated that the results were inconclusive [12].

Detection of minimum inhibitory concentration for vancomycin

On Muller-Hinton agar plates, *Enterococcus* isolates were lawn cultured, and vancomycin E-strip was placed on it. The minimum inhibitory concentration (MIC) was determined at the point where the ellipse crosses the MIC scale on the strip. As negative and positive controls, *E. faecalis* ATCC29212 and ATCC51299, respectively, were employed. Results were read into three categories. If MIC came 4 µg/mL or less, it was considered as sensitive, MIC of 8-16 µg/mL was considered as intermediate, and MIC of 32 µg/mL or more was considered as resistant [12].

Biofilm formation detection

Enterococci were screened for the biofilm formation by the microtiter plate method. BHI (brain heart infusion) broth with 1% glucose was used as culture medium to detect biofilm. On the basis of optical density reading by an ELISA reader, enterococci were categorized into biofilm non-producers and biofilm producers (strong, medium, and weak) [13-15].

## Results

A total of 164 *Enterococcus* species were isolated, of which *E. faecalis* was 60.97% and *E. faecium *was 39.02% from various clinical specimens, with p < 0.05 (significant value). The *Enterococcus* were maximum isolated from urine (28.05%), followed by pus (23.78%), and least from body fluids and stool (0.61%), as described in Table 1. Isolation of enterococci in different age group and gender were also done, where males were 62.8% and female were 37.19%. The age group of 21 to 30 years yielded maximum isolates (19.51%), whereas the age group of up to 10 years yielded the least isolates (6.10%), as shown in Table 2. *Enterococcus* species were maximum sensitive for teicoplanin (94.88%), followed by vancomycin (93.38%), linezolid (92.88%), and least sensitive for penicillin (17.19%) followed by norfloxacin (18.75%). Overall, *E. faecium* were less sensitive to tested antibiotics than *E. faecalis*, as shown in Table 3. Out of 164 isolates, 30 (18.29%) isolates were biofilm producers. *Enterococcus* isolated from pus samples were shown maximum (33.33) biofilm-producing capability followed by blood (16.67%) and urine (16.67%); on the other side, bronchoalveolar lavage fluid and pleural fluid sample isolates did not show any biofilm producer, as shown in Table 4. Biofilm producer *Enterococcus* isolates showed good sensitivity to linezolid (87.33%), followed by teicoplanin (86.43%) and vancomycin (79.64%), and least sensitivity to norfloxacin (6.79%). Overall, biofilm-producer isolates showed less sensitivity toward antibiotics compared to non-biofilm producers, as shown in Table 5. Comparative analysis between VRE and vancomycin-sensitive *Enterococcus *showed that males were more infected (72.72%), and the mean age of the patients was 33 years. *Enterococcus* *faecalis* showed more resistance (60.78%) toward vancomycin than *E. faecium *(39.21%), as shown in Table 6.

**Table 1:** Sample-wise distribution of Enterococcus (N = 164) BAL, bronchoalveolar lavage; ET, endotracheal; TT, transtracheal

Sample	Enterococcus faecalis		Enterococcus faecium		Total	
	N	%	N	%	N	%
BAL fluid	2	2	3	4.69	5	3.05
Blood	16	16	11	17.19	27	16.46
Catheter tip	2	2	2	3.13	4	2.44
ET secretion	11	11	2	3.13	13	7.93
Body fluid	1	1	0	0.00	1	0.61
Nasal swab	0	0	1	1.56	1	0.61
Pleural fluid	0	0	1	1.56	1	0.61
Pus	24	24	15	23.44	39	23.78
Sputum	7	7	4	6.25	11	6.71
Stool	1	1	0	0.00	1	0.61
TT aspirates	9	9	3	4.69	12	7.32
Urine	24	24	22	34.38	46	28.05
Vaginal swab	3	3	0	0.00	3	1.83
Total	100	60.97	64	39.02	164	100

**Table 2:** Gender-wise and age-wise distribution of patients

Age group	Male		Female		Total	
	N	%	N	%	N	%
Up to 10 years	8	7.77	2	3.28	10	6.10
11 to 20 years	12	11.65	8	13.11	20	12.20
21 to 30 years	17	16.50	15	24.59	32	19.51
31 to 40 years	17	16.50	6	9.84	23	14.02
41 to 50 years	14	13.59	13	21.31	27	16.46
51 to 60 years	18	17.48	8	13.11	26	15.85
Above 60 years	17	16.50	9	14.75	26	15.85
Total	103	62.80	61	37.19	164	100

**Table 3:** Antibiotic sensitivity patterns of Enterococcus species HLG, high-level gentamycin; HLS, high-level streptomycin

Antibiotics	Enterococcus faecalis (N = 100)		Enterococcus faecium (N = 64)		Mean %
	N	%	N	%	
Penicillin	20	20.00	11	17.19	18.60
Ampicillin	23	23.00	13	20.31	21.66
Vancomycin	93	93.00	60	93.75	93.38
Teicoplanin	96	96.00	60	93.75	94.88
Erythromycin	40	40.00	20	31.25	35.63
Tetracycline	69	69.00	45	70.31	69.66
Ciprofloxacin	63	63.00	38	59.38	61.19
Levofloxacin	62	62.00	38	59.38	60.69
Norfloxacin	16	16.00	12	18.75	17.38
Nitrofurantoin	21	21.00	15	23.44	22.22
Rifampin	66	66.00	41	64.06	65.03
Chloramphenicol	76	76.00	42	65.63	70.82
Linezolid	92	92.00	60	93.75	92.88
HLG	37	37.00	23	35.94	36.47
HLS	49	49.00	37	57.81	53.41

**Table 4:** Sample-wise distribution of biofilm producer Enterococcus species BAL, bronchoalveolar lavage; ET, endotracheal; TT, transtracheal

Sample	Total	Biofilm	%
BAL fluid	5	0	0.00
Blood	27	5	16.67
Catheter tip	4	2	6.67
ET secretion	13	3	10.00
Fluid	1	0	0.00
Nasal swab	1	1	3.33
Pleural fluid	1	0	0.00
Pus	39	10	33.33
Sputum	11	1	3.33
Stool	1	0	0.00
TT aspiration	12	1	3.33
Urine	46	5	16.67
Vaginal swab	3	2	6.67
Total	164	30	18.29

**Table 5:** Antibiotic sensitivity patterns among biofilm producers (N = 30) HLG, high-level gentamycin; HLS, high-level streptomycin

Antibiotics	Enterococcus faecalis (N = 17)		Enterococcus faecium (N = 13)		Mean %
	N	%	N	%	
Penicillin	4	23.53	1	7.69	15.61
Ampicillin	4	23.53	1	7.69	15.61
Vancomycin	14	82.35	10	76.92	79.64
Teicoplanin	15	88.24	11	84.62	86.43
Erythromycin	6	35.29	6	46.15	40.72
Tetracycline	13	76.47	8	61.54	69.01
Ciprofloxacin	7	41.18	7	53.85	47.52
Levofloxacin	7	41.18	7	53.85	47.52
Norfloxacin	1	5.88	1	7.69	6.79
Nitrofurantoin	2	11.76	2	15.38	13.57
Rifampin	8	47.06	7	53.85	50.46
Chloramphenicol	14	82.35	11	84.62	83.49
Linezolid	14	82.35	12	92.31	87.33
HLG	2	11.76	3	23.08	17.42
HLS	6	35.29	7	53.85	44.57

**Table 6:** Comparison analysis of VRE and VSE IPD, indoor patient; OPD, outdoor patient;VRE, vancomycin-resistant Enterococcus; VSE, vancomycin-sensitive Enterococcus

Variables	VRE (N =11)	%	VSE (N =153)	%
Sex				
Male	8	72.72	95	62.09
Female	3	27.27	58	37.90
Age mean years	33	-	39.88	-
Age distribution				
<10 years	1	9.09	9	5.88
11–50 years	8	72.72	94	61.43
>50 years	2	18.18	50	32.67
OPD/IPD				
OPD	5	45.45	51	33.33
IPD	6	54.54	102	66.66
Species				
Enterococcus faecalis	7	63.63	93	60.78
Enterococcus faecium	4	36.36	60	39.21

## Discussion

*Enterococcus faecalis *was the most common enterococci isolated in our study. These results are concordant with findings of Fernandes and Dhanashree, Jain et al., and Karmarkar et al. [16-18]. In contrast to our findings, *E. faecium *was identified as the dominating species by Telkar et al. [19]. In general, most of the research studies from all over the globe had concluded that *E. faecalis* was the dominant *Enterococcus *species, which have been involved in causing a wide range of diseases [20]. In the present study, the highest number of isolates were obtained from urine and pus samples, which were quite similar to the study by Kaur et al. [21], which showed almost similar results. Kaur et al. also found that the maximum numbers of Enterocooci were isolated in the age group of 21-30 years, which was in concordant with our results. On the other hand, Bhatt et al. [22] found that the majority of Enterocooci species were causing infections in patients aged 60 or above. The present study showed that 28% of *Enterococcus* species were isolated from urine samples. Different studies from different areas too concluded that urine was the most common sample yielding Enterococci, such as the studies by Mathur et al. [23], Karmarkar et al. [18], and Udo et al. [24], who reported rates of 49%, 50%, and 37% respectively. Vancomycin and teicoplanin resistance were detected in 6.7% and 4.87% of the isolates in the current investigation, respectively. There was not much significant difference in their resistance between *E. faecalis* and *E. faecium* for vancomycin and teicoplanin antibiotics. Additionally, it has been discovered in numerous investigations that although *E. faecium* makes up a far smaller proportion of clinical enterococcal isolates than *E. faecalis*, *E. faecium* was much more resistant to glycopeptides. Less than 2% of *E. faecalis* were found to be vancomycin-resistant in the research conducted by Deshpande et al. [25], which was a little lower compared to our findings; on the other hand, research published from south India showed that 52% of strains were vancomycin-resistant, and therefore the frequency and severity of glycopeptide resistance in this study were significantly higher [26]. This study showed that the total number of biofilm producers was 18.29%. The highest biofilm producers were isolated from pus specimens (33.33%). In contrast to our research, Khattak et al. [27] revealed that out of all enterococci, 33% of isolates were biofilm producers, and maximum biofilm producers belonged to pus samples, which were in concordance with the findings of the present study. Another study from India on enterococci demonstrated that 68% of isolates were biofilm formers, and among the biofilm producer, vancomycin resistance was 20.31% and teicoplanin resistance was 3.57% [28]. However, a study published by Shridhar and Dhanashree [29] reported no resistance to vancomycin, which was in contrast to our findings. There were a few limitations of the present study as we did not go for the detection of genes, which were associated with vancomycin resistance and biofilm production.

## Conclusions

Enterococci are one of the common infectious agents that are capable of causing a wide range of clinical complications. Treating enterococci infection is not that simple compared to other bacterial infections, as it is intrinsically resistant to many commonly used antibiotics such as cephalosporins and aminoglycosides. Moreover, the tendency to acquire drug resistance and the capability to produce biofilm make enterococcal disease management even worse. Hence, the availability of *Enterococcus* prevalence and their drug-resistant status in a particular hospital or area would give a better choice of antibiotics for better disease management and to control drug resistance in enterococci. The prevalence of VRE was found to be 6.7%, and antibiotic resistance was more in biofilm producers compared to non-biofilm producers in the present study. Teicoplanin, linezolid, and chloramphenicol are the drugs that have shown good activity against biofilm-producing and non-producing enterococci; therefore, they can be used in empirical treatment of enterococcal infections.
